# Epidemiological Evidence for Upper Respiratory Infections as a Potential Risk Factor for Meniere’s Disease: A Korean National Health Sample Cohort Study

**DOI:** 10.3390/microorganisms12102047

**Published:** 2024-10-10

**Authors:** Mi Jung Kwon, Ho Suk Kang, Joo-Hee Kim, Ji Hee Kim, Woo Jin Bang, Dae Myoung Yoo, Na-Eun Lee, Kyeong Min Han, Nan Young Kim, Hyo Geun Choi, Min-Jeong Kim, Eun Soo Kim

**Affiliations:** 1 Department of Pathology, Hallym University Sacred Heart Hospital, Hallym University College of Medicine, Anyang 14068, Republic of Korea; mulank99@hallym.or.kr; 2 Division of Gastroenterology, Department of Internal Medicine, Hallym University Sacred Heart Hospital, Hallym University College of Medicine, Anyang 14068, Republic of Korea; hskang76@hallym.or.kr; 3 Division of Pulmonary, Allergy, and Critical Care Medicine, Department of Medicine, Hallym University Sacred Heart Hospital, Hallym University College of Medicine, Anyang 14068, Republic of Korea; luxjhee@gmail.com; 4 Department of Neurosurgery, Hallym University Sacred Heart Hospital, Hallym University College of Medicine, Anyang 14068, Republic of Korea; kimjihee.ns@gmail.com; 5 Department of Urology, Hallym University Sacred Heart Hospital, Hallym University College of Medicine, Anyang 14068, Republic of Korea; yybbang@hallym.or.kr; 6 Hallym Data Science Laboratory, Hallym University College of Medicine, Anyang 14068, Republic of Korea; ydm@hallym.ac.kr (D.M.Y.); intriguingly@hallym.ac.kr (N.-E.L.); km.han@hallym.ac.kr (K.M.H.); 7 Laboratory of Brain and Cognitive Sciences for Convergence Medicine, Hallym University College of Medicine, Anyang 14068, Republic of Korea; 8 Hallym Institute of Translational Genomics and Bioinformatics, Hallym University Medical Center, Anyang 14068, Republic of Korea; honeyny@hallym.or.kr; 9 Suseo Seoul E.N.T. Clinic, 10, Bamgogae-ro 1-gil, Gangnam-gu, Seoul 06349, Republic of Korea; mdanalytics@naver.com; 10 Department of Radiology, Hallym University Sacred Heart Hospital, Hallym University College of Medicine, Anyang 14068, Republic of Korea; drkmj@hallym.or.kr

**Keywords:** upper respiratory infections, Meniere’s disease, national sample cohort study, risk factor, big data analysis

## Abstract

Meniere’s disease (MD) is a chronic inner ear disorder characterized by tinnitus, ear fullness, episodic vertigo, and fluctuating hearing loss, which significantly impacts quality of life and poses management challenges. Recent evidence suggests that upper respiratory infections (URIs) may contribute to MD’s onset. This study examines the potential link between URIs and MD using data from the Korean National Health Insurance Service-National Sample Cohort (2002–2019). We analyzed 19,721 individuals with MD and 78,884 matched controls, adjusting for demographic factors and comorbidities using propensity score matching. Our results showed that individuals with a URI within one year prior to the index date exhibited a 2.01-fold greater likelihood of developing MD (95% confidence interval [CI] = 1.91–2.11, *p* < 0.001), while those with URIs within two years demonstrated a 1.54-fold higher probability (95% CI = 1.50–1.59, *p* < 0.001). Furthermore, we found that even remote URIs occurring up to two years before the index date significantly increased the risk of developing MD, underscoring the need for long-term patient follow-up. Overall, our study suggests that individuals with a history of URI may have an elevated risk of developing MD over multiple time frames, regardless of demographic or health profiles.

## 1. Introduction

Meniere’s disease is an inner ear condition with an unknown cause, marked by endolymphatic hydrops and recurring episodes of vertigo that can last from minutes to hours, as well as symptoms such as tinnitus, worsening hearing loss, and a sensation of ear fullness [1]. These symptoms significantly affect quality of life and pose challenges in management [1]. It predominantly affects white females, particularly those in their 40s and 50s, with its incidence increasing with age [2,3]. The frequency and incidence of Meniere’s disease vary across ethnicities and geographic regions worldwide, with rates ranging from 3 to 513 cases per 100,000 annually, depending on the studies [4,5]. These variations may be attributed to differences in diagnostic criteria between countries, environmental factors such as diet or climate, and genetic predispositions in different populations [4,5]. However, the incidence and complex etiology of Meniere’s disease remains difficult to determine due to the limited availability of comprehensive epidemiological data [6].

Depending on the studies, the frequency and incidence of Meniere’s disease differ across ethnicities and geographic regions worldwide, with rates ranging from 3 to 513 cases per 100,000 individuals annually [4,5], with non-whites generally exhibiting a lower prevalence. The incidence and complex etiology remain difficult to determine due to limited epidemiological data [6]. For example, in Japan, the prevalence is remarkably low, ranging from 5.8 to 37 per 100,000 adults [7]. In stark contrast, South Korea has experienced a significant rise from 30.02 to 118.48 per 100,000 between 2013 and 2017 [8]. This dramatic rise is attributed to factors like population aging and rapid lifestyle changes, making Meniere’s disease a growing public health concern in the region [8]. Nonetheless, there is no established consensus on how to treat Meniere’s disease [6]. This trend may highlight the need for a deeper insight into Meniere’s disease etiology to develop effective prevention and management strategies for this progressively debilitating condition.

Meniere’s disease is believed to have a multifactorial etiology, with various factors potentially contributing to its development, including genetic predisposition, environmental influences, viral infections, allergies, and dysfunctions of the immune and autonomic nervous systems [1,9]. Endolymphatic hydrops, characterized by excessive endolymph buildup in the inner ear causing damage to ganglion cells, is a key pathological feature of Meniere’s disease. It can result from various causes, including infectious factors [3,10]. 

Recent studies indicate that upper respiratory infections (URIs) may contribute to the development of Meniere’s disease [9,11,12], warranting further investigation into this possible link. URIs, which include a range of illnesses such as the common cold, sinusitis, and influenza, are some of the most common infectious diseases affecting humans [13]. Research has increasingly linked viruses such as influenza, cytomegalovirus, varicella-zoster, Epstein-Barr, and severe acute respiratory syndrome coronavirus 2 (COVID-19) to Meniere’s disease [14,15], demonstrating that endolymphatic sac cells are highly susceptible to viral infections that disrupt vestibular function [11]. Notably, a prospective study detected viral DNA associated with URIs in the endolymphatic sac of patients with Meniere’s disease [16], potentially suggesting a URI viral impact on inner ear function. Interestingly, during the COVID-19 pandemic, while total outpatient audiological visits decreased, the incidence of first-time diagnoses of Meniere’s disease rose significantly to 3.2% in 2020, up from 1.3% in 2018 and 1.2% in 2019 [15]. Additionally, a retrospective study found that 27.6% of 87 COVID-19 patients experienced otologic symptoms during infection, including dizziness (16.1%), hearing loss (11.5%), tinnitus (11.5%), imbalance (11.5%), vertigo (4.6%), ear drainage (1.1%), and facial nerve palsy (1.1%) [14]. A meta-analysis of 14 case-control studies identified a 3.65-fold association (95% CI 1.27–10.46) between cytomegalovirus infection and Meniere’s disease compared to controls [17]. However, these studies lacked a priori power analysis and the sample size was fewer than 20 [17]. The reporting on the health status of both the study and control group participants was incomplete [17], indicating the need for further validation.

Despite growing concern over the past decade, the connection between URIs and Meniere’s disease has not been thoroughly explored in large, population-based studies. To date, there is limited primary research specifically exploring this connection. This research aims to bridge this gap by analyzing a comprehensive nationwide cohort from 2002 to 2019, including 19,721 individuals diagnosed with Meniere’s disease and 78,884 matched controls. The study leverages a meticulously curated database, ensuring balanced demographics between the groups and minimizing potential confounders. This approach facilitates a clearer understanding of the potential influence of URIs on Meniere’s disease, setting the stage for targeted interventions that could mitigate its impact on public health.

## 2. Materials and Methods

### 2.1. Data Source and Participant Selection

This study, approved by the Ethics Committee of Hallym University, Anyang, South Korea, complied with all ethical guidelines, with the requirement for informed consent waived by the Institutional Review Board (IRB No. 2022-10-008). 

We utilized data from the Korean National Health Insurance Service-National Sample Cohort (KNHIS-NSC) [18], which includes records of 1,137,861 individuals and 219,673,817 medical billing codes from 2002 to 2019. This cohort represents about 2.2% of the entire Korean population, systematically sampled to ensure national representation, and followed for 17 years [19]. The KNHIS-NSC uses the International Classification of Diseases, 10th revision (ICD-10) codes for standardized disease diagnosis and healthcare data management.

A nested case-control design was employed to examine the relationship between medical history, exposure, and outcomes. Participants newly diagnosed with Meniere’s disease (ICD-10 code: H810) between 2002 and 2019 (n = 20,838), with at least two treatments and audiometric exams, were selected. Those diagnosed with Meniere’s disease in 2002 and 2003 were removed to allow a two-year washout period, resulting in 1117 removals. Individuals without a diagnosis during this period were included as controls (n = 1,117,023), but 20,785 controls with any history of Meniere’s disease were omitted. 

**Figure 1:**
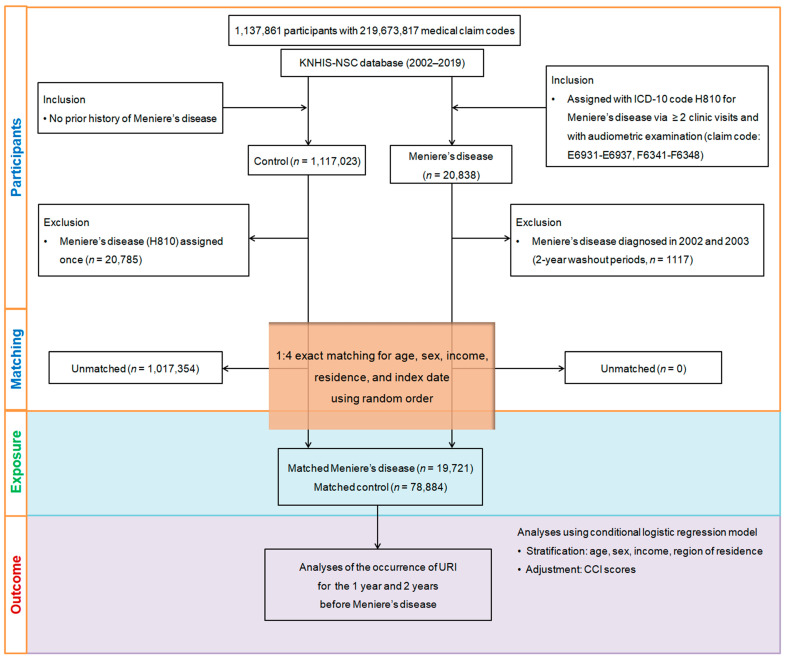
A visual overview of the participant selection and matching process in the study. Starting with the initial pool of 1,137,861 individuals in the Korean National Health Insurance Service-National Sample Cohort (KNHIS-NSC) database, a meticulous selection process resulted in 19,721 patients diagnosed with Meniere’s disease being matched with 78,884 control participants. Matching was based on age, sex, income, and region of residence.

Propensity score matching was applied to balance demographic and clinical characteristics (age, sex, income, region) between Meniere’s disease patients and controls, minimizing selection bias. Each Meniere’s disease patient’s index date was matched to the control group, resulting in the exclusion of 1,017,354 controls and a final sample of 19,721 Meniere’s disease patients matched with 78,884 controls in a 1:4 ratio. We then analyzed the history of URIs within 1-year and 2-year periods prior to the index date for both groups (Figure 1).

During data cleaning, patients with missing data or only a single Meniere’s disease diagnosis were excluded. A two-year washout period was applied to avoid including pre-existing Meniere’s disease cases. Covariates for adjustment were selected based on relevant literature identifying potential confounders between Meniere’s disease and URI incidence. Stratification was performed based on age (18 categories), sex, income (5 categories), and region (urban/rural). A 1:4 propensity score matching process was conducted, with unmatched patients being excluded. Covariates closest to the index date were used, and conditional logistic regression was applied to control for confounding factors, while the Charlson Comorbidity Index (CCI) was used to assess the overall disease burden.

### 2.2. Exposure (Upper Respiratory Infection) and Outcome (Meniere’s Disease)

To enhance the accuracy of the analysis and minimize false-positive cases, we included only patients who had received treatment for upper respiratory infections (URIs) on multiple medical visits. These cases were identified using specific ICD-10 codes: J00 for acute nasopharyngitis, J02 for acute pharyngitis, and J069 for acute upper respiratory infection. The number of clinic or hospital visits for URI-related treatments was documented for each participant up to one day before the 1-year and 2-year periods preceding the index date. This comprehensive approach was designed to ensure the reliability of the observed association between URIs and the study outcomes [20].

Meniere’s disease cases were identified using the ICD-10 code H810. Participants included in the study were those who had been treated for Meniere’s disease at least twice and had undergone an audiometric examination (claim codes: E6931-E6937, F6341-F6348) [10].

### 2.3. Covariates

Participants were divided into 10 age groups, each spanning 5 years, and further categorized into five income levels, from level 1 (lowest) to level 5 (highest). For residential classification, areas were grouped into 16 counties based on administrative boundaries and then further divided into urban (the 7 largest cities in Korea, each with populations over one million) or rural (regions with populations under one million) areas [21].

To assess the overall disease burden, the CCI was used, which considers 17 different comorbid conditions. Each participant was assigned a total CCI score based on the number and severity of these conditions, with scores ranging from 0 (indicating no comorbidities) to 29 (indicating multiple severe comorbidities) [22]. 

### 2.4. Statistical Analyses

Standardized differences were used to compare baseline characteristics between groups. A covariate was deemed to be well-balanced if its absolute standardized difference was ≤0.20 [23]. For any covariate with an absolute standardized difference greater than 0.20 after matching, we made further adjustments using multivariable logistic regression analysis [23]. 

To calculate the odds ratios (ORs) for the association between URIs and Meniere’s disease, along with 95% confidence intervals (CIs), conditional logistic regression was applied to matched groups based on age, sex, income, and region of residence. These analyses included both crude (unadjusted) and adjusted models (adjusted for CCI scores). URI treatments were examined by dividing them into two categories: those within 1 year before the index date and those within 2 years before the index date. We calculated 95% CIs for each group. Subgroup analyses were conducted using conditional logistic regression based on age, sex, income, and region of residence, while unconditional logistic regression was used for CCI scores.

Statistical significance was assessed using the exact two-tailed tests used for the hypothesis testing, with *p*-values less than 0.05 considered significant. All statistical analyses were performed using SAS version 9.4 (SAS Institute Inc., Cary, NC, USA).

## 3. Results

The study included 19,721 individuals diagnosed with Meniere’s disease and 78,884 matched controls, with matching based on age, sex, income level, and region of residence. The demographic characteristics of both groups were identical, with standardized differences of 0.00 for these variables. The mean CCI scores were similar between the groups, with a standardized difference of 0.03, indicating no significant disparity in comorbid burden. Notably, the Meniere’s disease group had a higher average number of URI-related clinical visits within both 1 year and 2 years compared to the controls (Table 1).

To enhance the reliability of our findings, we performed a detailed analysis of URI histories at both the 1-year and 2-year intervals prior to the index date. The results consistently showed a strong positive association between a history of URIs and the development of Meniere’s disease within both time frames. The OR for the 1-year analysis was 2.01 (95% CI = 1.91–2.11, *p* < 0.001), indicating a 2.01-fold increased likelihood of developing Meniere’s disease. Similarly, for the 2-year analysis, the OR was 1.54 (95% CI = 1.50–1.59, *p* < 0.001), reflecting a 54% greater likelihood compared to the control group (Table 2).

**Figure 2:**
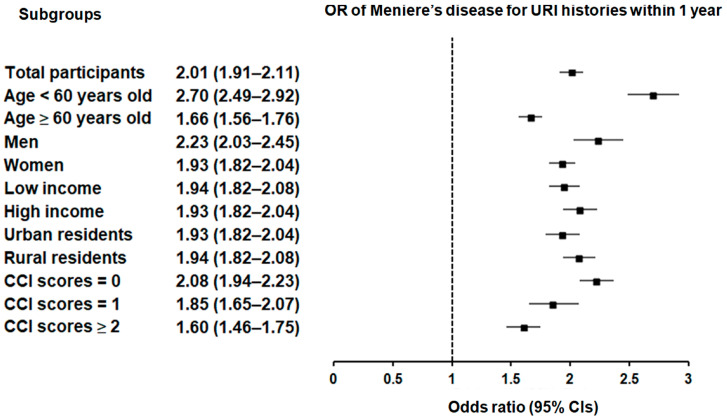
Forest plots illustrating the adjusted odds ratio and corresponding 95% confidence intervals (CIs) for demographic, socioeconomic, and comorbid factors in relation to upper respiratory infections (URIs) for incident Meniere’s disease when participants are diagnosed and treated with URI within 1 year before the index date.

**Figure 3:**
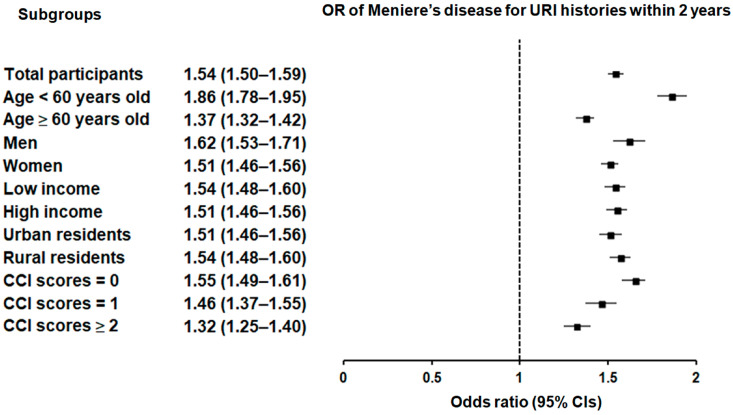
Forest plots illustrating the adjusted odds ratio and corresponding 95% confidence intervals (CIs) for demographic, socioeconomic, and comorbid factors in relation to upper respiratory infections (URIs) for incident Meniere’s disease when participants are diagnosed and treated with URI within 2 years before the index date.

Subgroup analyses confirmed that the significant association between a history of URI within both 1 year and 2 years prior to the index date and a heightened likelihood of Meniere’s disease remained consistent across all groups, independent of age, sex, income, region of residence, or CCI scores (Figure 2 and Figure 3).

## 4. Discussion

Meniere’s disease significantly impairs daily activities and social interaction due to symptoms like vertigo and progressive hearing loss, even with available treatments. The link between URIs and Meniere’s disease has not been extensively studied in large, population-based research. Most existing studies have focused on individual symptoms of Meniere’s disease [24] or its association with viral infections [17], rather than directly examining URIs as a whole. 

In our extensive nationwide cohort study, we examined data from two different time periods to determine a robust association between URIs and Meniere’s disease. Our findings offer, to the best of our knowledge, the first nationwide-level epidemiological evidence that a history of URIs may significantly elevate the likelihood of developing Meniere’s disease. Specifically, individuals with a URI within one year prior to the index date experienced a 2.01-fold elevated likelihood of developing Meniere’s disease (95% CI = 1.91–2.11, *p* < 0.001). This risk remained elevated at a 1.54-fold (95% CI = 1.50–1.59, *p* < 0.001) likelihood of developing Meniere’s disease for URIs occurring within two years prior to the index date, reinforcing the robust link between these two conditions. The significant relationship remained consistent across all subgroups, including income, the comorbid burden, age, sex, and place of residence. This uniformity across different subgroups may suggest that preventive strategies targeting URIs could have broad applicability in reducing the risk of Meniere’s disease. During the COVID-19 pandemic, although there was an increase in outpatient clinic cancellations for the follow-up of audiological diseases overall, outpatients with Meniere’s disease reported significantly fewer cancellations [25], indicating a strong necessity for continuous care even amidst public health crises [25]. Given the consistency of our results across diverse demographic and socioeconomic backgrounds, which suggests that a history of URI may be a standalone risk element for Meniere’s disease and potentially serve as a trigger or contributing factor to its onset, the early identification and management of URIs may be clinically important as a strategy to mitigate the onset of Meniere’s disease.

Although the acute symptoms of URIs are usually mild and brief, growing evidence indicates that they can have more significant and lasting impacts on human health than previously understood [13], potentially resulting in severe complications like asthma, congestive heart failure, pneumonia, and acute exacerbations of chronic obstructive pulmonary disease [12,26,27]. Additionally, URIs can trigger the production of cytokines and chemokines, which may cause vascular endothelial cell injury and contribute to conditions like stroke, hypertension, and myocardial infarction [12,28]. These inflammatory responses and vascular endothelial damage could also exacerbate the pathophysiological processes underlying Meniere’s disease [9,24]. Our study may further support this link, revealing that even remote URIs occurring up to two years before the index date may significantly increase the likelihood of developing Meniere’s disease; the adjusted OR for URIs within one year prior to the index date is 2.01, indicating a more than twofold increase in risk. This risk remains elevated over a two-year period, with an adjusted OR of 1.54, still reflecting a significant association. These findings align with a prospective study that detected viral DNA in the endolymphatic sacs of ten Meniere’s disease patients: seven with varicella-zoster virus, four with Epstein-Barr virus, and one with cytomegalovirus [16]. Despite these findings, the lack of elevated serum antibody titers before endolymphatic sac surgery suggests that the viral DNA may be latent and inactive, indicating that previous viral infections could reach the endolymphatic sac and potentially contribute to Meniere’s disease development [16]. 

Another study found that 20% of patients had a delay of over five years between hearing loss and vertigo onset, with gadolinium-enhanced magnetic resonance imaging (MRI) showing endolymphatic hydrops in 90% of Meniere’s disease cases [3]. Potential risk factors for Meniere’s disease, including infections, may act as triggers that disrupt the biochemical and physiological functions of the inner ear or neural structures in the brain, contributing to the gradual onset of symptoms over time [29]. These disruptions can potentially alter fluid homeostasis, leading to endolymphatic hydrops and affecting innerear function [29]. Of note, recurrences of Meniere’s disease are also more frequent within the first 12 to 13 months following the initial gentamicin treatment, indicating the need for long-term patient follow-up of more than two years [30].

The exact mechanisms linking URIs to subsequent Meniere’s disease remain unclear. Meniere’s disease and URIs appear to share common risk factors, including infection, inflammation, immune response, and underlying vascular conditions, as well as genetic predispositions that play a role in the development of both diseases [9,12,24,26,31]. Viruses or bacteria from a cold or sore throat can potentially migrate to the middle or inner ear, causing fluid buildup, and conditions like otitis media may worsen Meniere’s symptoms [26,29]. This spread typically occurs through the Eustachian tube, which connects the middle ear to the upper respiratory tract [11]. In this context, gentamicin, an aminoglycoside antibiotic widely used in clinical practice, has been employed in the treatment and prophylaxis of over 40 clinical conditions, including respiratory tract infections and Meniere’s disease. Clinical trials have demonstrated that the intratympanic application of gentamicin is a relatively safe and effective treatment for reducing vertigo attacks, aural fullness, and hearing disturbances associated with Meniere’s disease [30,32].

Moreover, viruses typically involved in URIs—such as coronaviruses, rhinovirus, adenoviruses, and influenza—may cause vascular endothelium injury, to which the inner ear is particularly sensitive [11]. This damage can disrupt the production of vasodilators (nitric oxide and prostacyclin) and increase proinflammatory cytokines (such as tumor necrosis factor-α, interleukin-6, and interleukin-1) due to changes in blood clotting and fibrinolysis, heightening the risk of thrombotic complications that might impair inner ear function and contribute to Meniere’s disease symptoms [9], especially sensorineural hearing loss [24]. Also, viral infections from URIs could initiate or exacerbate Meniere’s disease through inflammatory responses or autoimmune reactions [9].

In addition, both URIs and Meniere’s disease appear to share certain genetic loci associated with genetic susceptibility, involving multiple biochemical pathways [9,12]. Although the shared genetics between URIs and Meniere’s disease have not been well studied, URI-associated non-synonymous variants in the genes *NFKB1*, *IL7R*, and *TNFRSF13B*—classified as Mendelian immune disorder genes—suggest shared heritability with autoimmune conditions and immune deficiency, particularly through the TNFR2 pathway [12]. In Meniere’s disease, TWEAK binding to Fn14 activates both the canonical and non-canonical NF-κB pathways, which play key roles in immunity and immune-mediated disorders [33]. The non-canonical pathway relies on signals from TNFR family members, including Fn14, TNFR2, BAFFR, CD40, LTβR, and RANK [9]. These insights suggest that variants in immune response genes may affect the susceptibility to both URIs and Meniere’s disease. 

Several limitations of this study should be noted. As an observational and retrospective analysis, our study cannot establish a definitive causal relationship between URI history and Meniere’s disease, nor does it explore the underlying mechanisms involved. Additionally, the focus was solely on the Korean population, and the study primarily relied on Korean health insurance data for assessing exposure, which means there may be unmeasured confounding factors that were not considered. This focus could limit the generalizability of our findings to other populations and demographic groups. Moreover, the KNHIS-NSC database does not provide detailed information on the severity of URIs or Meniere’s disease, family medical history, or genetic background. Consequently, our analysis could not account for these missing details, which may affect the consideration of potential unmeasured confounders. The Korean National Health Insurance Service database has been widely utilized in research. A study validating the primary diagnostic codes of major clinical outcomes calculated the positive predictive value, sensitivity, and specificity of these codes using hospital medical records as the gold standard [34]. For major clinical outcomes, the positive predictive value was over 90%, indicating the favorable reliability of the Korean National Health Insurance Service database’s diagnostic codes. However, it is important to note that validation studies specifically focused on the diagnostic accuracy of Meniere’s disease or URI are currently lacking.

Despite its limitations, this study has notable strengths. It utilizes a large, representative cohort of 19,721 Meniere’s disease patients and 78,884 controls drawn from a comprehensive nationwide healthcare database, which enhances its generalizability and precision. This extensive dataset, covering a wide cross-section of the Korean adult population, provides thorough medical histories across the country, allowing for detailed analysis. Although Meniere’s disease typically has a lower incidence in non-white populations [7,8], our study is the first to explore the link between a history of URIs and the occurrence of Meniere’s disease using a nationwide cohort in Korea. The robustness of this research is further supported by adjustments for socioeconomic factors, comorbidities, and other potential confounding variables. Given the higher prevalence of Meniere’s disease among females, older adults, and those with higher household incomes, as well as its association with various comorbidities [35], we applied propensity score matching to reduce confounding and selection bias, ensuring better comparability between the patient and control groups.

## 5. Conclusions

This study demonstrated that individuals with a history of URI may be at a higher risk of developing Meniere’s disease across multiple time frames and irrespective of demographic or comorbidity profiles. The study further indicates the importance of long-term patient follow-up, as even URIs occurring up to two years before the index date significantly elevated the likelihood of developing MD, highlighting the pervasive and long-term impact of URIs on Meniere’s disease risk. The consistent findings across various subgroups may underscore a strong association between URIs and Meniere’s disease, suggesting the potential universal applicability of preventive strategies targeting URIs in the context of Meniere’s disease. Our study contributes to the growing body of evidence as the first nationwide-level epidemiologic study to establish a significant link between URIs and Meniere’s disease, emphasizing the importance of addressing URIs in disease prevention efforts.

## Figures and Tables

**Table 1:** General characteristics of participants.

Characteristics	Meniere’s Disease	Control	Standardized Difference
Age (y), n (%)			0.00
0–4	3 (0.02)	12 (0.02)	
5–9	31 (0.16)	124 (0.16)	
10–14	135 (0.68)	540 (0.68)	
15–19	387 (1.96)	1548 (1.96)	
20–24	471 (2.39)	1884 (2.39)	
25–29	596 (3.02)	2384 (3.02)	
30–34	838 (4.25)	3352 (4.25)	
35–39	1178 (5.97)	4712 (5.97)	
40–44	1415 (7.18)	5660 (7.18)	
45–49	1718 (8.71)	6872 (8.71)	
50–54	1947 (9.87)	7788 (9.87)	
55–59	2113 (10.71)	8452 (10.71)	
60–64	1979 (10.03)	7916 (10.03)	
65–69	2046 (10.37)	8184 (10.37)	
70–74	1965 (9.96)	7860 (9.96)	
75–79	1572 (7.97)	6288 (7.97)	
80–84	901 (4.57)	3604 (4.57)	
85+	426 (2.16)	1704 (2.16)	
Sex, n (%)			0.00
Male	5998 (30.41)	23,992 (30.41)	
Female	13,723 (69.59)	54,892 (69.59)	
Income, n (%)			0.00
1 (lowest)	4062 (20.60)	16,248 (20.60)	
2	2674 (13.56)	10,696 (13.56)	
3	3109 (15.76)	12,436 (15.76)	
4	4257 (21.59)	17,028 (21.59)	
5 (highest)	5619 (28.49)	22,476 (28.49)	
Region of residence, n (%)			0.00
Urban	8414 (42.67)	33,656 (42.67)	
Rural	11,307 (57.33)	45,228 (57.33)	
CCI score (mean, SD)	0.76 (1.40)	0.71 (1.45)	0.03
The number of upper respiratory infections (mean, SD)			
within 1 year	2.09 (3.62)	1.34 (3.02)	0.23
within 2 year	4.10 (6.35)	2.69 (5.13)	0.24

Abbreviations: CCI, Charlson Comorbidity Index; SD, standard deviation.

**Table 2:** Crude and adjusted odds ratios of Meniere’s disease in relation to the treatments for URI within two different time periods from the index date, with subgroup analyses according to age, sex, income, region of residence, and CCI scores.

Characteristics		Odd Ratios for Meniere’s Disease (95% Confidence Interval)			
		Crude ^†^	*p*	Adjusted ^‡^	*p*
From the index date to before the 1-year period					
Total participants (n = 98,605)		2.01 (1.92–2.11)	<0.001 *	2.01 (1.91–2.11)	<0.001 *
	Age < 60 years old (n = 54,160)	2.72 (2.51–2.94)	<0.001 *	2.70 (2.49–2.92)	<0.001 *
	Age ≥ 60 years old (n = 44,445)	1.66 (1.56–1.76)	<0.001 *	1.66 (1.56–1.76)	<0.001 *
	Male (n = 29,990)	2.24 (2.04–2.45)	<0.001 *	2.23 (2.03–2.45)	<0.001 *
	Female (n = 68,615)	1.93 (1.82–2.05)	<0.001 *	1.93 (1.82–2.04)	<0.001 *
	Low income group (n = 49,225)	1.95 (1.82–2.09)	<0.001 *	1.94 (1.82–2.08)	<0.001 *
	High income group (n = 49,380)	2.08 (1.94–2.23)	<0.001 *	2.08 (1.94–2.23)	<0.001 *
	Urban residents (n = 42,070)	1.93 (1.79–2.08)	<0.001 *	1.93 (1.79–2.08)	<0.001 *
	Rural residents (n = 56,535)	2.08 (1.95–2.21)	<0.001 *	2.07 (1.94–2.21)	<0.001 *
	CCI scores = 0 (n = 67,493)	2.21 (2.07–2.36)	<0.001 *	2.22 (2.08–2.37)	<0.001 *
	CCI scores = 1 (n = 14,086)	1.85 (1.65–2.07)	<0.001 *	1.85 (1.65–2.07)	<0.001 *
	CCI scores ≥ 2 (n = 17,026)	1.59 (1.45–1.74)	<0.001 *	1.60 (1.46–1.75)	<0.001 *
From the index date to before the 2-year period					
Total participants (n = 98,605)		1.54 (1.50–1.59)	<0.001 *	1.54 (1.50–1.59)	<0.001 *
	Age < 60 years old (n = 54,160)	1.87 (1.79–1.96)	<0.001 *	1.86 (1.78–1.95)	<0.001 *
	Age ≥ 60 years old (n = 44,445)	1.37 (1.32–1.42)	<0.001 *	1.37 (1.32–1.42)	<0.001 *
	Male (n = 29,990)	1.62 (1.54–1.71)	<0.001 *	1.62 (1.53–1.71)	<0.001 *
	Female (n = 68,615)	1.51 (1.46–1.57)	<0.001 *	1.51 (1.46–1.56)	<0.001 *
	Low income group (n = 49,225)	1.54 (1.48–1.60)	<0.001 *	1.54 (1.48–1.60)	<0.001 *
	High income group (n = 49,380)	1.55 (1.49–1.61)	<0.001 *	1.55 (1.49–1.61)	<0.001 *
	Urban residents (n = 42,070)	1.51 (1.45–1.58)	<0.001 *	1.51 (1.45–1.58)	<0.001 *
	Rural residents (n = 56,535)	1.57 (1.51–1.63)	<0.001 *	1.57 (1.51–1.63)	<0.001 *
	CCI scores = 0 (n = 67,493)	1.64 (1.58–1.70)	<0.001 *	1.65 (1.58–1.71)	<0.001 *
	CCI scores = 1 (n = 14,086)	1.46 (1.37–1.55)	<0.001 *	1.46 (1.37–1.55)	<0.001 *
	CCI scores ≥ 2 (n = 17,026)	1.32 (1.25–1.39)	<0.001 *	1.32 (1.25–1.40)	<0.001 *

Abbreviations: URI, Upper respiratory infection; CCI, Charlson Comorbidity Index. * Significance at *p* < 0.05. ^†^ Crude models were stratified by age, sex, income, and region of residence. ^‡^ Adjusted for CCI scores.

## Data Availability

All data are available from the database of the National Health Insurance Sharing Service (NHISS) at https://nhiss.nhis.or.kr/ (accessed on 1 March 2023). NHISS allows access to all of this data for any researcher who agrees to follow the research ethics guidelines, subject to a processing charge. If you want to access the data from this article, you can download it from the website after agreeing to follow the research ethics guidelines.
